# Development and validation of novel immune-inflammation-based clinical predictive nomograms in HER2-negative advanced gastric cancer

**DOI:** 10.3389/fonc.2023.1185240

**Published:** 2023-09-06

**Authors:** Yan Yang, Yu Shao, Junjun Wang, Qianqian Cheng, Hanqi Yang, Yulong Li, Jing Liu, Yangyang Zhou, Zhengguang Zhou, Mingxi Wang, Baoan Ji, Jinghao Yao

**Affiliations:** ^1^ Department of Medical Oncology, The First Affiliated Hospital of Bengbu Medical College, Bengbu, China; ^2^ Department of Radiation Oncology, The First Affiliated Hospital of Bengbu Medical College, Bengbu, China; ^3^ Department of Clinical Laboratory, The First Affiliated Hospital of Bengbu Medical College, Bengbu, China; ^4^ Department of Surgical Oncology, The First Affiliated Hospital of Bengbu Medical College, Bengbu, China; ^5^ Department of Cancer Biology, Mayo Clinic, Jacksonville, FL, United States; ^6^ Department of Oncology, The First Affiliated Hospital of Anhui Medical University, Hefei, China

**Keywords:** nomogram, predictive model, VEGFA, HER2-negative, advanced gastric cancer

## Abstract

**Purpose:**

To explore the predictive value of multiple immune-inflammatory biomarkers including serum VEGFA and systemic immune-inflammation index (SII) in HER2-negative advanced gastric cancer (AGC) and establish nomograms for predicting the first-line chemotherapeutic efficacy, progression-free survival (PFS) and overall survival (OS) of patients with this fatal disease.

**Methods:**

From November 2017 to April 2022, 102 and 34 patients with a diagnosis of HER2-negative AGC at the First Affiliated Hospital of Bengbu Medical College were enrolled as development and validation cohorts, respectively. Univariate and multivariate analyses were performed to evaluate the clinical value of the candidate indicators. The variables were screened using LASSO regression analysis. Predictive models were developed using significant predictors and are displayed as nomograms.

**Results:**

Baseline VEGFA expression was significantly higher in HER2-negative AGC patients than in nonneoplastic patients and was associated with malignant serous effusion and therapeutic efficacy (all p<0.001). Multivariate analysis indicated that VEGFA was an independent predictor for first-line therapeutic efficacy and PFS (both p<0.01) and SII was an independent predictor for first-line PFS and OS (both p<0.05) in HER2-negative AGC patients. The therapeutic efficacy model had an R^2^ of 0.37, a Brier score of 0.15, and a Harrell’s C-index of 0.82 in the development cohort and 0.90 in the validation cohort. The decision curve analysis indicated that the model added more net benefits than VEGFA assessment alone. The PFS/OS models had Harrell’s C-indexes of 0.71/0.69 in the development cohort and 0.71/0.62 in the validation cohort.

**Conclusion:**

The established nomograms integrating serum VEGFA/SII and commonly available baseline characteristics provided satisfactory performance in predicting the therapeutic efficacy and prognosis of HER2-negative AGC patients.

## 1 Introduction

Gastric cancer (GC) is the fifth most diagnosed cancer and the fourth leading cause of cancer-related death worldwide (1). Currently, for human epidermal growth factor receptor 2 (HER2)-negative GC, chemotherapy remains the cornerstone of systemic therapy for patients with advanced disease despite the emergence of immune checkpoint inhibitors (ICIs) (2, 3). Due to the highly heterogeneous and malignant nature of GC (4), patient response to chemotherapy varies greatly from person to person, with survival varying widely; most patients with advanced disease die within 12 months of diagnosis (5). Therefore, it is critical to identify new approaches to predict the efficacy and prognosis of chemotherapy to make more appropriate treatment decisions and improve survival in patients with advanced gastric cancer (AGC).

The immune-inflammatory response is widely accepted to play important roles in tumor initiation, invasion, angiogenesis, and metastasis (6, 7). Angiogenesis is one of the hallmarks of malignancy and contributes significantly to the growth, invasion, and metastatic spread of cancer (8). As a major marker of angiogenesis, vascular endothelial growth factor A (VEGFA) expression is associated with the treatment response (9) and prognosis of various malignancies (10, 11). In addition, other immune-inflammatory biomarkers such as systemic immune-inflammation index (SII) (12), c-reactive protein/albumin ratio (CAR) (13); nutritional markers such as body mass index (BMI) (14), hemoglobin (Hb) (15), albumin/globulin ratio (AGR) (16), and lipoprotein cholesterol (17); and the classic tumor marker carcinoembryonic antigen (CEA) (18) were associated with GC or other malignancies. Since the predictive efficacy of a single indicator is limited, there is a need to rationally evaluate diverse indicators of treatment efficacy and prognosis and to select appropriate parameters to establish predictive models that can comprehensively assess treatment effectiveness and provide individualized treatment for patients with malignant tumors.

Currently, most of the predictive models for GC are diagnostic and prognostic models (19, 20) and are based on retrospective studies; some of them are based on gene expression and are difficult to be applied clinically (21, 22). This prospective, single-center, cohort study aimed to predict the effectiveness of first-line chemotherapy and the treatment outcome for HER2-negative AGC using clinicopathologic features and laboratory hematologic indicators to provide convenient and general predictive models for the treatment of AGC and to improve clinical decision making and patient−physician communication.

## 2 Materials and methods

### 2.1 Patient selection

From November 2017 to April 2022, we prospectively enrolled 102 and 34 patients who attended the First Affiliated Hospital of Bengbu Medical College for primary treatment of recurrent or metastatic GC as development and validation cohorts. The inclusion criteria were as follows: patients with histologically confirmed HER2-negative AGC; patients who had not received antitumor therapy against recurrent or metastatic GC; patients who were not suitable for or were unwilling to undergo surgery or radiation therapy; and patients with target lesions that could be evaluated for efficacy. The exclusion criteria were as follows: patients with a combination of other tumors or subtypes; patients with severe cardiac, hepatic, or renal diseases; and patients with severe bleeding or infectious diseases. Moreover, 108 patients with nonneoplastic disease without bleeding or infectious diseases were retrospectively enrolled as a control for the development cohort and 15 HER2-positive AGC patients treated with first-line trastuzumab combined with chemotherapy from September 2019 to April 2022 were retrospectively enrolled as a HER2-positive cohort. The present study was reported in accordance with the STROBE (23) and TRIPOD (24) guidelines as much as possible. This study was conducted in accordance with the Declaration of Helsinki and was approved by the Institutional Review Board of the First Affiliated Hospital of Bengbu Medical College (BYYFY-2017KY09). Informed consent was obtained from all patients or their family members.

### 2.2 Bioinformatics analysis

VEGFA mRNA levels in several types of cancer, including GC, were analyzed using the Tumor Immune Estimation Resource (TIMER2.0) database with The Cancer Genome Atlas (TCGA) data (http://timer.cistrome.org/). The GDC dataset containing the gene expression profiles (HTSeq-Counts) of 27 pairs of GC tissues and their matched normal tissues was downloaded from The Cancer Genome Atlas stomach adenocarcinoma (TCGA-STAD, https://xenabrowser.net/). To ensure uniformity across the data, the RNA-seq profiles were transformed into TPM values, and the formula log2 (TPM+1) was used for normalization. GC samples from TCGA-STAD were selected for survival analysis. Using the Kaplan−Meier plotter database (25), the prognostic role of VEGFA mRNA expression in HER2-negative GC patients at stage IV was analyzed (Probe ID: 211527_at_x).

### 2.3 Predictive variables

Clinicopathological information such as sex, age, height, weight, Eastern Cooperative Oncology Group performance status (ECOG PS), diagnosis pattern (including recurrent or primary cancer), histology [including adenocarcinoma (AC) and signet-ring cell carcinoma (SRC)], position of the primary lesion, number and sites of metastases, comorbidities (such as malignant serous effusion), and laboratory indicators [such as VEGFA, CEA, total protein (TP), albumin (ALB), Hb, C-reactive protein (CRP), low-density lipoprotein cholesterol (LDL-C), and high-density lipoprotein cholesterol (HDL-C)] were collected at baseline for each patient. Composite indicators were calculated based on the following formulas: AGR=ALB/(TP-ALB), BMI=height/weight^2^, CAR=CRP/ALB, LHR=LDL-C/HDL-C, and SII=platelet count × neutrophil count/lymphocyte count.

### 2.4 Detection of VEGFA and other laboratory indicators

Five milliliter samples of nonanticoagulated peripheral venous blood and 3 ml of EDTA-K2 anticoagulated peripheral venous blood were collected from each patient at baseline. The nonanticoagulated peripheral venous blood was centrifuged at 3000 rpm for 10 min to separate the serum, and the supernatant was quickly frozen and stored at -20°C. VEGFA detection was performed using a Weigao JR-1 Chemiluminescent Immunoassay Analyzer and Vascular Endothelial Growth Factor Assay Kit (chemiluminescence, Shandong Weigao Group Medical Polymer Co., Ltd., Weihai, China) according to the manufacturer’s instructions. A Sysmex XE-2100 automatic blood analyzer (Sysmex Corporation, Kobe, Japan) was used for hematology analysis. According to the standards of laboratory SOP documents and reagent instructions, CEA measured by chemiluminescence microparticle immunoassay was detected using an Abbott I-2000 chemiluminescence immunoanalyzer, and all reagents, calibration and quality control products were obtained from Abbott Laboratories Trading Co., Ltd. (Shanghai, China). ALB/TP (measured by colorimetric assay), LDL-C/HDL-C (measured by homogeneous enzyme colorimetric assay), and CRP (measured by immunoturbidimetric assay) were detected using a Roche Cobas 8000 C 701 automatic biochemical analyzer, and all reagents, calibration and quality control products were obtained from Roche Diagnostics Co., Ltd. (Shanghai, China). All the test technicians were professionally trained and did not have access to clinical data.

### 2.5 Therapeutic regimens

All patients received at least one cycle of first-line chemotherapy according to the NCCN guidelines for gastric cancer (version 5. 2017) (26). Two-drug regimens (fluorouracil or capecitabine/oxaliplatin or cisplatin; fluorouracil/irinotecan; paclitaxel or docetaxel/cisplatin) were mainly used in this study. Monotherapy (fluorouracil; capecitabine; paclitaxel or docetaxel) was administered to patients with a poor ECOG PS. The doses of the regimens were decided by physicians according to the actual condition of the patients.

### 2.6 Chemotherapeutic efficacy assessment

Chemotherapeutic efficacy was evaluated according to the Response Evaluation Criteria in Solid Tumors (RECIST) version 1.1 (27) every 2 cycles or when necessary by 2 independent senior physicians. The optimal therapeutic efficacy was recorded as complete response (CR), partial response (PR), stable disease (SD) (all of which indicated effective treatment), or progressive disease (PD) (which indicated ineffective treatment). The following formulas were applied to determine the objective response rate (ORR) and the disease control rate (DCR): ORR = (CR + PR)/(CR + PR + SD + PD); DCR = (CR + PR + SD)/(CR + PR + SD + PD).

### 2.7 Follow-up and outcomes

Patients were followed-up by telephone or hospital review until disease progression, death, or loss to follow-up. PFS was calculated from the start of first-line chemotherapy until disease progression or death. Overall survival (OS) was calculated from the start of first-line chemotherapy until death. The outcome measures used in this study were chemotherapeutic efficacy (non-PD or PD), PFS, and OS.

### 2.8 Statistical analysis

R for Windows (version 4.2.0, https://www.r-project.org/) was used as the primary tool for data analysis and graphing. All statistical tests were two-sided, and *P<*0.05 was considered to indicate statistical significance. The age data followed a normal distribution and were reported as the means. Other continuous data did not follow a normal distribution and were reported as medians [interquartile range (IQR)]. Continuous variables of two groups of independent samples were compared using Student’s t test (normal distribution) or Wilcoxon test (nonnormal distribution). Continuous variables of more than two groups of independent samples were compared using the Kruskal−Wallis test (nonnormal distribution). Correlations between continuous variables were analyzed using Spearman’s correlation test. Cutoffs for Hb and AGR were determined based on clinical significance. The optimal cutoffs of VEGFA, CEA, SII, LHR, and CAR in therapeutic efficacy analyses were determined according to the maximum Youden’s index. Predictive variables associated with therapeutic efficacy were initially screened using univariate logistic regression analysis. Variables with *P<*0.05 were included in the multivariate logistic regression analysis, and odds ratios (ORs) and corresponding 95% confidence intervals (CIs) were calculated. A receiver operating characteristic (ROC) curve was used to evaluate the accuracy of the prediction of therapeutic efficacy, and the area under the curve (AUC) was calculated. Optimal cutoffs for VEGFA, CEA, SII, LHR and CAR in survival analyses were determined according to the method of maximally selected rank and statistics. Prognostic variables associated with PFS and OS were initially screened using a univariate Cox regression analysis. Variables with *P<*0.05 were included in the multivariate Cox regression analysis, and hazard ratios (HRs) and corresponding 95% confidence intervals (CIs) were calculated. Survival analyses were performed using the Kaplan−Meier method, and comparisons of survival between the two groups were performed using the log-rank test.

Least absolute shrinkage and selection operator (LASSO) logistic and Cox regression analyses were performed to screen for candidate variables associated with therapeutic efficacy and prognosis, respectively. The number of events per independent variable (EPV) of each model followed the 10EPV principle as much as possible (28). Four variables were selected to fit the predictive model of therapeutic efficacy using the logistic regression model and five variables were selected to fit the prognostic models of PFS and OS using the Cox regression model. The models’ overall performance was assessed using R^2^ or Brier scores, and discrimination was evaluated using ROC curves and Harrell’s C-index. The models were internally validated by a bootstrap resampling method (1000 random samples were drawn from the original dataset and replaced between each resample step) (29, 30). Calibration was assessed using a calibration curve or the Hosmer and Lemeshow goodness of fit test statistic. Decision curve analysis (DCA) was performed to estimate the clinical utility of the model predicting therapeutic efficacy by calculating the net benefit for a range of threshold probabilities. Finally, a nomogram and a dynamic nomogram for each model were established.

## 3 Results

### 3.1 Relationship between VEGFA expression and either GC development or GC patient prognosis according to the TCGA and Kaplan−Meier Plotter databases

**Figure 1:**
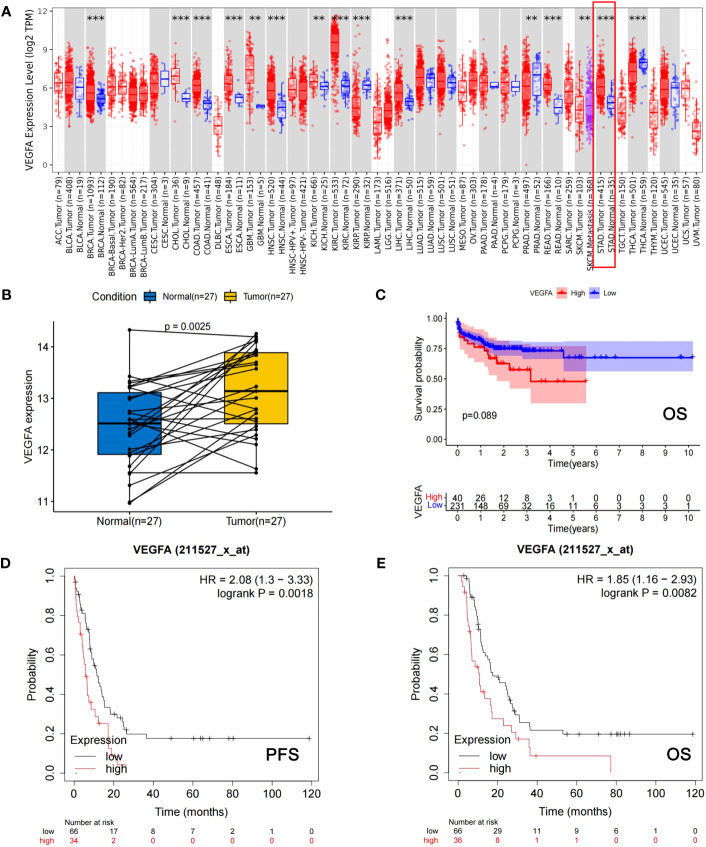
The mRNA expression level of VEGFA is significantly associated with the development of GC and the prognosis of GC patients. **(A)** The mRNA expression levels of VEGFA in cancerous tissues versus normal tissues among different types of cancer, including GC (indicated by red box). **(B)** The mRNA expression levels of VEGFA in cancerous versus corresponding normal tissues of GC patients. **(C)** Kaplan−Meier survival curves of OS for GC patients from TCGA database with different mRNA expression levels of VEGFA. **(D, E)** Kaplan−Meier survival curves of PFS and OS of HER2-negative AGC patients from the Kaplan−Meier Plotter database with different mRNA expression levels of VEGFA. **: p<0.01, ***: p<0.001.

The VEGFA mRNA level was significantly higher in GC tissues (n=415) than in normal gastric tissue (n=35) (*p*<0.001, **Figure 1A**) and matched tissues (n=27, p=0.003, **Figure 1B**) in the TCGA database. Survival analyses according to the mRNA expression levels of VEGFA in GC using the TCGA database showed that patients with high mRNA expression levels of VEGFA had a trend toward shorter OS (n=271, p=0.089, **Figure 1C**) in all GC populations, and high VEGFA expression was significantly associated with shorter PFS (n=100, HR: 2.08, 95% CI: 1.30-3.33, p=0.002, **Figure 1D**) and OS (n=102, HR: 1.85, 95% CI: 1.16-2.93, p=0.008, **Figure 1E**) in patients with HER2-negative GC at stage IV from Kaplan−Meier plotter database.

### 3.2 Diagnostic value of baseline serum VEGFA between patients with HER2-negative AGC and nonneoplastic disease

**Figure 2:**
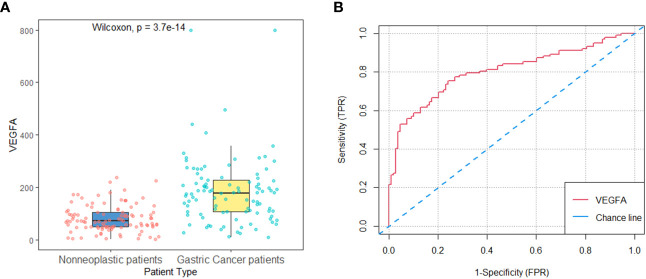
Baseline serum VEGFA in patients with nonneoplastic disease versus HER2-negative AGC in the development cohort. **(A)** Baseline serum VEGFA levels were significantly higher in HER2-negative AGC patients (n=102) than in patients with nonneoplastic disease (n=108) (*P*<0.001). **(B)** The AUC of the ROC curve indicating the ability of baseline VEGFA to distinguish between patients with nonneoplastic disease and HER2-negative AGC patients was 0.80 (95% CI: 0.74-0.86, *P<*0.001).

The HER2-negative AGC patients (n=102) in the development cohort included 62 (60.8%) men and 40 (39.2%) women, and 71 (65.7%) men and 37 (34.3%) women were included in the nonneoplastic group (n=108). The mean age did not differ significantly between the two groups (62.7 vs. 59.7 years, p=0.084). The baseline serum VEGFA level was significantly higher in AGC patients than in patients with nonneoplastic disease (178.7 (106.5-226.7) vs. 71.7 (49.3-105.9) pg/ml, p*<*0.001, **Figure 2A**). The AUC of the ROC curve indicting the ability of baseline VEGFA to distinguish between HER2-negative AGC and nonneoplastic patients was 0.80 (95% CI: 0.74-0.86, p*<*0.001, **Figure 2B**). With a cutoff value of 106.3 pg/ml, VEGFA had a sensitivity of 75.5%, a specificity of 75.9%, a positive likelihood ratio (+LR) of 3.14, and a negative likelihood ratio (−LR) of 0.32.

### 3.3 Differences in baseline serum VEGFA, therapeutic efficacy and survival between HER2-negative and HER2-positive AGC patients

**Figure 3:**
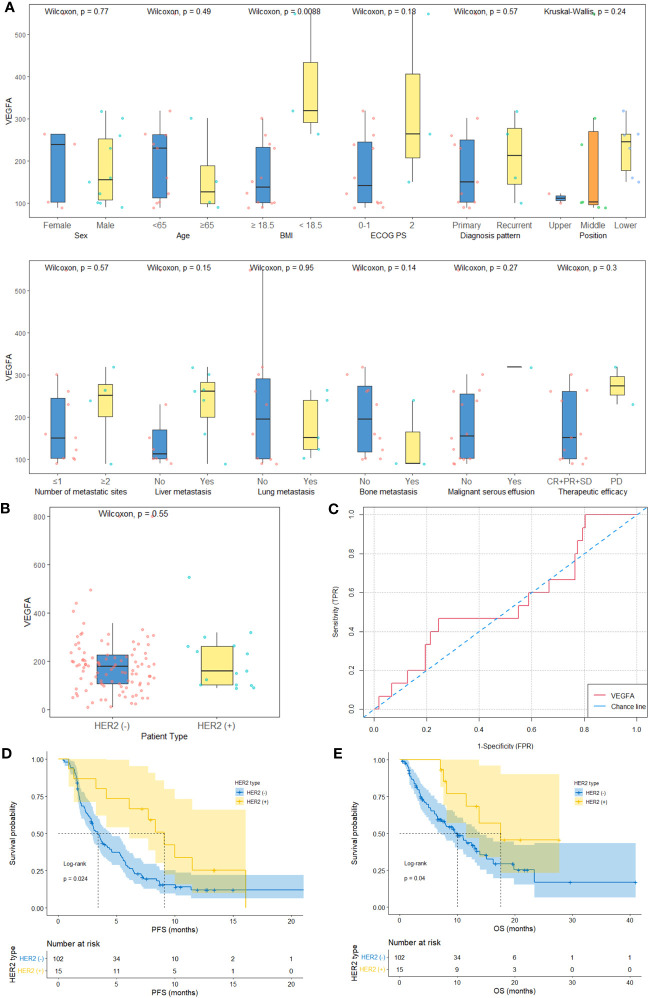
Baseline serum VEGFA and survival in HER2-negative AGC versus HER2-positive AGC patients. **(A)** The baseline characteristics and relationships between baseline serum VEGFA and the clinicopathological characteristics of HER2-positive AGC patients (histology plot is not shown). **(B)** Baseline serum VEGFA levels were comparable between HER2-negative AGC patients (n=102) and HER2-positive AGC patients (n=15) (*P*=0.549). **(C)** The AUC of the ROC curve indicating the ability of baseline VEGFA to distinguish between HER2-negative and HER2-positive AGC patients was 0.45 (95% CI: 0.29-0.62, *P=*0.565). **(D, E)** Kaplan−Meier survival curves of PFS and OS of HER2-negative and HER2-positive AGC patients.

The median follow-up times of the development, validation and HER2-positive cohorts were 13.1 months (95% CI: 10.3-16.6 months), 20.9 months (95% CI: 13.4-NA months) and 18.2 months (95% CI: 13.4-NA months), respectively. There are 6 and 1 patients were lost to OS follow-up in the development and validation cohorts, respectively. The baseline characteristics of the HER2-positive (n=15) and HER2-negative cohorts (development cohort, n=102) and their relationships to baseline serum VEGFA levels are shown in **Table S1**, **Figure 3A** and **Table 1**, respectively. The HER2-negative AGC patients (n=102) in the development cohort included 62 (60.8%) men and 40 (39.2%) women, and 10 (66.7%) men and 5 (33.3%) women were included in the HER2-positive cohort (n=15). The mean age between the two groups did not show a significant difference (62.7 vs. 57.5 years, p=0.126). The baseline serum VEGFA levels were similar between HER2-negative and HER2-positive AGC patients (178.7 (106.5-226.7) vs. 160.5 (102.7-262.8) pg/ml, p=0.549, **Figure 3B**). The AUC of the ROC curve indicting the ability of baseline VEGFA to distinguish between patients with HER2-negative and HER2-positive AGC was 0.45 (95% CI: 0.29-0.62, p=0.565, **Figure 3C**). The ORR and DCR were significantly higher in the HER2-positive cohort than in the HER2-negative cohort (ORR: 0.67 vs. 0.23; DCR: 0.87 vs. 0.68). The median PFS (mPFS) and median OS (mOS) were significantly longer in the HER2-positive cohort than in the HER2-negative cohort (mPFS: 9.1 vs. 3.4 months, p=0.024, **Figure 3D**; mOS: 17.5 vs. 10.0 months, p=0.040, **Figure 3E**).

**Table 1:** Relationship between baseline serum VEGFA and clinicopathological characteristics of HER2-negative AGC patients.

Characteristics	Groups	Development cohort				Validation cohort			
		N (%)	Median (IQR)	Statistics	p-value	N (%)	Median (IQR)	Statistics	p-value
Sex	Female	40 (39.2%)	185.54 (107.82-257.76)	1454	0.143	11 (32.4%)	190.05 (85.22-483.91)	130	0.912
	Male	62 (60.8%)	161.46 (104.71-209.46)			23 (67.6%)	168.53 (118.33-283.53)		
Age	<65	54 (52.9%)	180.19 (105.33-232.91)	1357.5	0.683	18 (52.9%)	165.01 (123.54-239.19)	137	0.823
	≥65	48 (47.1%)	161.46 (106.65-216.60)			16 (47.1%)	204.25 (99.16-304.49)		
BMI (kg/m^2^)	≥ 18.5	81 (79.4%)	174.66 (104.05-225.98)	773	0.524	33 (97.1%)	168.53 (101.87-287.41)	11	0.61
	< 18.5	21 (20.6%)	192.55 (130.19-226.88)			1 (2.9%)	230.52 (230.52-230.52)		
ECOG PS	0-1	63 (61.8%)	174.66 (92.46-234.94)	1097.5	0.369	22 (64.7%)	165.01 (93.75-270.26)	106	0.358
	2	39 (38.2%)	186.13 (116.49-220.05)			12 (35.3%)	196.66 (141.44-674.57)		
Diagnosis pattern	Primary	56 (54.9%)	183.14 (116.92-215.31)	1410.5	0.412	18 (52.9%)	164.55 (100.73-222.84)	127	0.569
	Recurrent	46 (45.1%)	154.48 (81.33-247.10)			16 (47.1%)	199.52 (111.88-304.49)		
Histology	AC	84 (82.4%)	177.12 (103.57-213.68)	651.5	0.361	26 (76.5%)	186.51 (106.76-332.10)	131	0.282
	SRC	18 (17.6%)	191.89 (111.60-301.24)			8 (23.5%)	163.94 (107.32-193.85)		
Position	Upper	39 (38.2%)	160.49 (79.17-213.82)	1.39	0.499	12 (35.3%)	176.66 (111.88-288.23)	1.56	0.459
	Middle	36 (35.3%)	178.60 (111.79-239.64)			12 (35.3%)	186.51 (151.95-680.73)		
	Lower	27 (26.5%)	187.72 (109.72-207.95)			10 (29.4%)	141.47 (91.04-229.07)		
Number of metastatic sites	≤1	81 (79.4%)	178.30 (95.00-234.92)	795	0.649	27 (79.4%)	168.53 (118.33-236.30)	70	0.307
	≥2	21 (20.6%)	181.26 (130.19-201.95)			7 (20.6%)	345.91 (96.46-695.73)		
Liver metastasis	No	73 (71.6%)	178.30 (97.44-236.87)	1047	0.935	22 (64.7%)	165.01 (123.54-230.07)	119	0.652
	Yes	29 (28.4%)	181.26 (110.27-207.93)			12 (35.3%)	222.68 (91.04-425.86)		
Lung metastasis	No	88 (86.3%)	178.71 (100.96-234.93)	605	0.919	26 (76.5%)	196.66 (123.54-285.47)	126	0.383
	Yes	14 (13.7%)	171.84 (134.70-206.84)			8 (23.5%)	124.98 (91.04-213.80)		
Bone metastasis	No	91 (89.2%)	181.26 (106.59-234.94)	616	0.215	30 (88.2%)	165.01 (93.75-270.26)	32	0.142
	Yes	11 (10.8%)	118.93 (94.52-191.24)			4 (11.8%)	448.12 (206.86-680.73)		
Malignant serous effusion^#^	No	60 (58.8%)	146.60 (79.53-190.37)	678	<0.001***	19 (55.9%)	161.49 (88.91-217.88)	89	0.066
	Yes	42 (41.2%)	211.72 (148.96-303.38)			15 (44.1%)	228.71 (125.62-575.23)		
Therapeutic efficacy	CR+PR+SD	69 (67.6%)	144.95 (85.44-201.38)	627	<0.001***	21 (61.8%)	121.45 (86.78-168.53)	24	<0.001***
	PD	33 (32.4%)	206.04 (185.01-280.61)			13 (38.2%)	290.68 (230.52-665.73)		

***: p<0.001.

^#^ Malignant serous effusion was diagnosed based on ascites cytology or clinical symptoms if abdominocentesis was contraindicated or cytology result was unavailable.

### 3.4 Relationships between baseline serum VEGFA and other clinicopathological characteristics of HER2-negative AGC patients in the development and validation cohorts

Considering that HER2-positive AGC patients show relatively better treatment responses and survival than HER2-negative AGC patients and that these patients represent a small proportion of the GC patient population according to epidemiological studies, we focused on HER2-negative AGC patients for an in-depth analysis. A flowchart of the study is shown in **Figure S1A**. The baseline characteristics and relationships between baseline serum VEGFA levels and the clinicopathological characteristics of patients in the development (n=102) and validation cohorts (n=34) are shown in **Table 1**. Regarding clinicopathological characteristics, baseline VEGFA was significantly associated with malignant serous effusion and therapeutic efficacy in the development cohort (both p*<*0.001, **Figure S1B**, **Table 1**) and only therapeutic efficacy in the validation cohort (p*<*0.001, **Table 1**). In addition, baseline VEGFA was not significantly correlated with any of the other assessed laboratory indicators in the development cohort (all p>0.05, **Figure S1C**).

### 3.5 Relationship between predictive variables and the therapeutic efficacy of first-line chemotherapy in HER2-negative AGC patients

**Figure 4:**
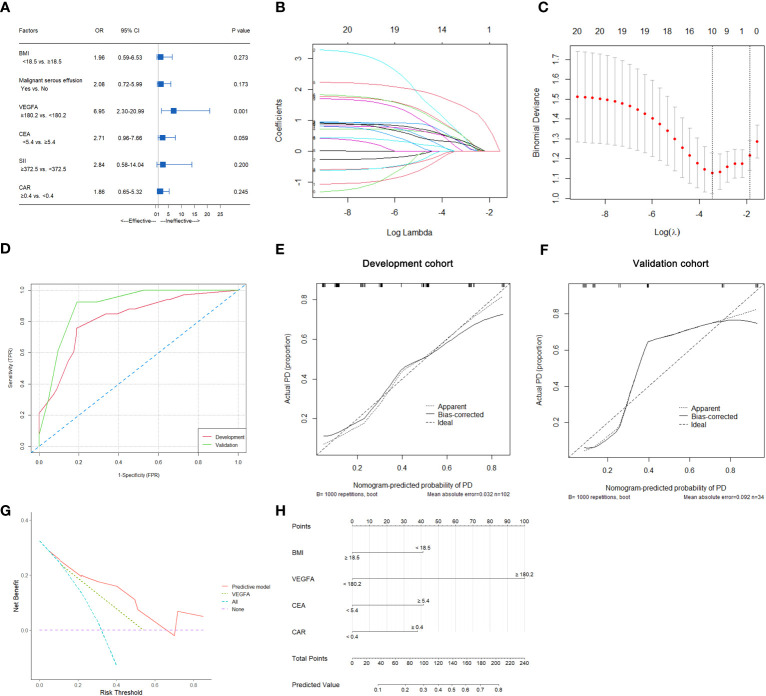
Predictive variable screening and construction, evaluation and presentation of a predictive model of therapeutic efficacy in HER2-negative AGC patients. **(A)** Forest plot showing the multivariate logistic analysis of the predictive variables that were significantly associated with therapeutic efficacy in the univariate logistic analyses. **(B, C)** Predictive variables were screened using the LASSO regression model via 10-fold cross-validation based on minimum criteria. The dotted vertical line in C was plotted at the value selected using 10-fold cross-validation in C. **(D)** ROC curves of the predictive model for predicting the therapeutic efficacy of HER2-negative AGC patients in the development and validation cohorts. **(E)** Calibration plot of the predictive model for HER2-negative AGC in the development cohort. **(F)** Calibration plot of the predictive model for HER2-negative AGC in the validation cohort. **(G)** The DCA for the predictive model and VEGFA alone in the development cohort. **(H)** Nomogram to predict the probability of PD at first evaluation in patients with HER2-negative AGC.

In this study, 33 of 102 patients with therapeutic efficacy data were confirmed to have PD. As shown in **Table S2**, univariate analysis suggested that BMI (p=0.032), malignant serous effusion (p=0.007), VEGFA (p<0.001), CEA (p=0.031), SII (p=0.044) and CAR (p=0.013) were significantly associated with therapeutic efficacy in the development cohort. Multivariate analysis indicated that only VEGFA [≥180.2 vs. <180.2, odds ratio (OR): 6.95, 95% CI: 2.30-20.99, p=0.001] was an independent predictive factor for therapeutic efficacy in HER2-negative AGC patients (**Table S2**, **Figure 4A**).

### 3.6 Development of nomograms for predicting therapeutic efficacy in HER2-negative AGC patients with first-line chemotherapy

In all 102 HER2-negative AGC patients with therapeutic efficacy data, variables were screened according to the LASSO logistic regression model (**Figures 4B, C**), clinical significance, and recommendations from oncology experts, and BMI, VEGFA, CEA and CAR were selected to fit a logistic regression model for predicting the therapeutic efficacy of PD at first evaluation. The model had an R^2^ of 0.37 and a Brier score of 0.15. The discriminatory efficiency of the model was evaluated by ROC analysis, which indicated that the model achieved a Harrell’s C-index of 0.82 (95% CI: 0.73-0.91) before calibration and 0.79 after calibration with internal validation using bootstrap resampling (1,000 repetitions) in the development cohort and 0.90 (95% CI: 0.79-1.00) in the validation cohort (**Figure 4D**). The calibration curves of the model in the development and validation cohorts are illustrated in **Figures 4E, F**; the Hosmer−Lemeshow test statistics indicated nonsignificant differences between sample types in the development cohort (p=0.370). The DCA for the model is presented in **Figure 4G**, which indicated that the model adds more net benefit than VEGFA alone and the “treat all” or “treat none” strategies. Finally, the nomogram and web-based dynamic nomogram of the therapeutic efficacy predictive model are presented in **Figure 4H** and https://cancerprediction.shinyapps.io/efficacy_prediction_of_her2-agc/. The calculation formula for the model is presented on the webpage of the dynamic nomogram.

### 3.7 Relationship between predictive variables and PFS after first-line treatment in HER2-negative AGC patients

**Figure 5:**
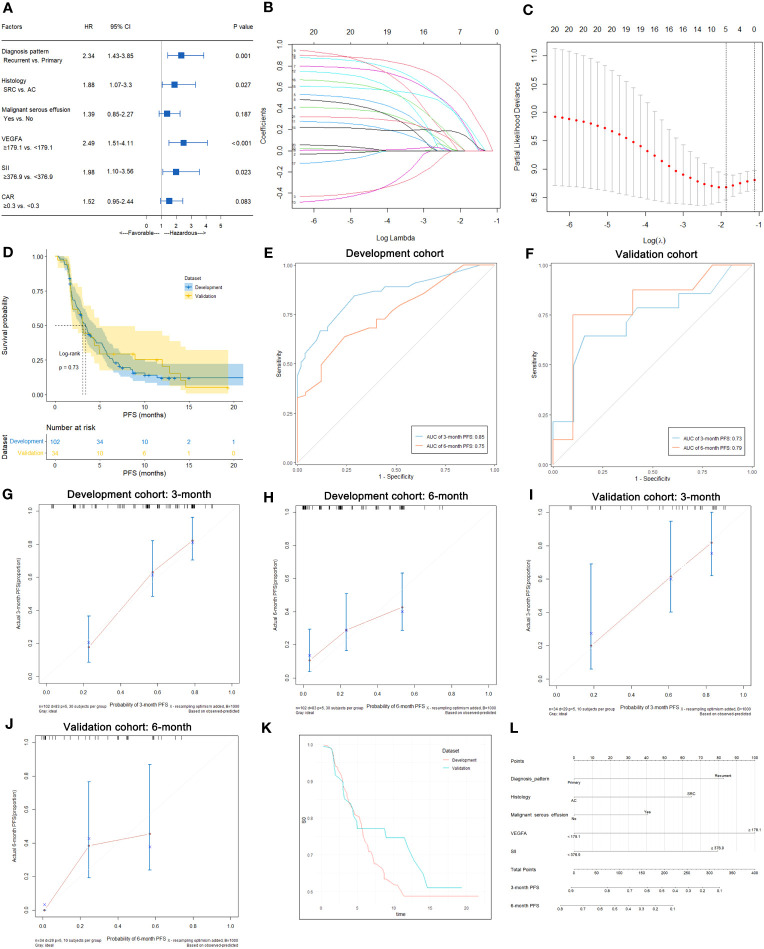
Predictive variable screening and construction, evaluation and presentation of the PFS predictive model in HER2-negative AGC patients. **(A)** Forest plot showing the multivariate Cox analysis of PFS with the predictive variables that were significantly associated with PFS in the univariate Cox analyses. **(B, C)** Predictive variables were screened using the LASSO regression model via 10-fold cross-validation based on minimum criteria. The dotted vertical line in C was plotted at the value selected using 10-fold cross-validation in **(C)**. **(D)** Kaplan−Meier survival curves of PFS of HER2-negative AGC patients in the development and validation cohorts. **(E)** Time-dependent ROC curves of the PFS predictive model at 3 and 6 months for HER2-negative AGC in the development cohort. **(F)** Time-dependent ROC curves of the PFS predictive model at 3 and 6 months for HER2-negative AGC in the validation cohort. **(G, H)** Calibration plot of the PFS predictive model at 3 and 6 months for HER2-negative AGC in the development cohort. **(I, J)** Calibration plot of the PFS predictive model at 3 and 6 months for HER2-negative AGC in the validation cohort. **(K)** Baseline survival curves of PFS of HER2-negative AGC patients in the development and validation cohorts. **(L)** Nomogram to predict the probability of 3-month or 6-month PFS in patients with HER2-negative AGC.

**Figure 6:**
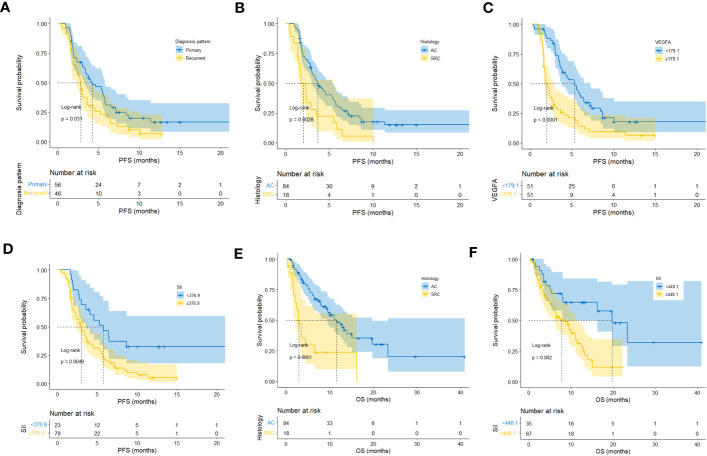
Kaplan−Meier plots of patients stratified by each independent prognostic factor of first-line PFS and OS. **(A–D)** Kaplan−Meier plots of patients stratified by each independent prognostic factor of first-line PFS. **(E, F)** Kaplan−Meier plots of patients stratified by each independent prognostic factor of OS.

In this study, 83 of 102 patients with complete follow-up data reached the PFS endpoint. As shown in **Table S3**, univariate analysis suggested that the diagnosis pattern (p=0.033), histology (p=0.004), malignant serous effusion (p=0.003), VEGFA (p<0.001), SII (p=0.006) and CAR (p=0.045) were significantly associated with PFS in patients with HER2-negative AGC. Multivariate analysis indicated that diagnosis pattern (recurrent vs. primary, HR: 2.34, 95% CI: 1.43-3.85, p=0.001), histology (signet-ring cell carcinoma vs. adenocarcinoma, HR: 1.88, 95% CI: 1.07-3.3, p=0.027), VEGFA (≥179.1 vs. <179.1, HR: 2.49, 95% CI: 1.51-4.11, p<0.001) and SII (≥376.9 vs. <376.9, HR: 1.98, 95% CI: 1.10-3.56, p=0.023) were independent prognostic factors for PFS in HER2-negative AGC patients (**Table S3**, **Figure 5A**). Kaplan−Meier plots of patients stratified by each independent prognostic factor are shown in **Figures 6A–D**.

### 3.8 Development of nomograms for predicting PFS after first-line treatment in HER2-negative AGC patients

In all 102 HER2-negative AGC patients with complete follow-up data, variables were screened according to the LASSO Cox regression model (**Figures 5B, C**), clinical significance, and recommendations from oncology experts, and diagnosis pattern, histology, malignant serous effusion, VEGFA and SII were selected to fit a Cox regression model for predicting PFS that met the proportional hazards assumption. The mPFS was similar between the development and validation cohorts (p=0.730, **Figure 5D**). The model had Harrell’s C-indexes of 0.71 and 0.70 after calibration with internal validation using bootstrap resampling (1,000 repetitions) in the development cohort and 0.71 in the validation cohort. The time-dependent ROC curves and calibration curves of the 3-month and 6-month PFS prediction in the development (AUC: 0.85 and 0.75, respectively) and validation cohorts (AUC: 0.73 and 0.79, respectively) are illustrated in **Figures 5E–J**. The baseline survival curves of PFS were similar in both development and validation cohorts (**Figure 5K**). The nomogram and web-based dynamic nomogram of the predictive PFS model are presented in **Figure 5L** and https://cancerprediction.shinyapps.io/pfs_prediction_of_her2-agc/. The calculation formula for the PFS model is presented on the webpage of the dynamic nomogram.

### 3.9 Relationship between predictive variables and OS of HER2-negative AGC patients

**Figure 7:**
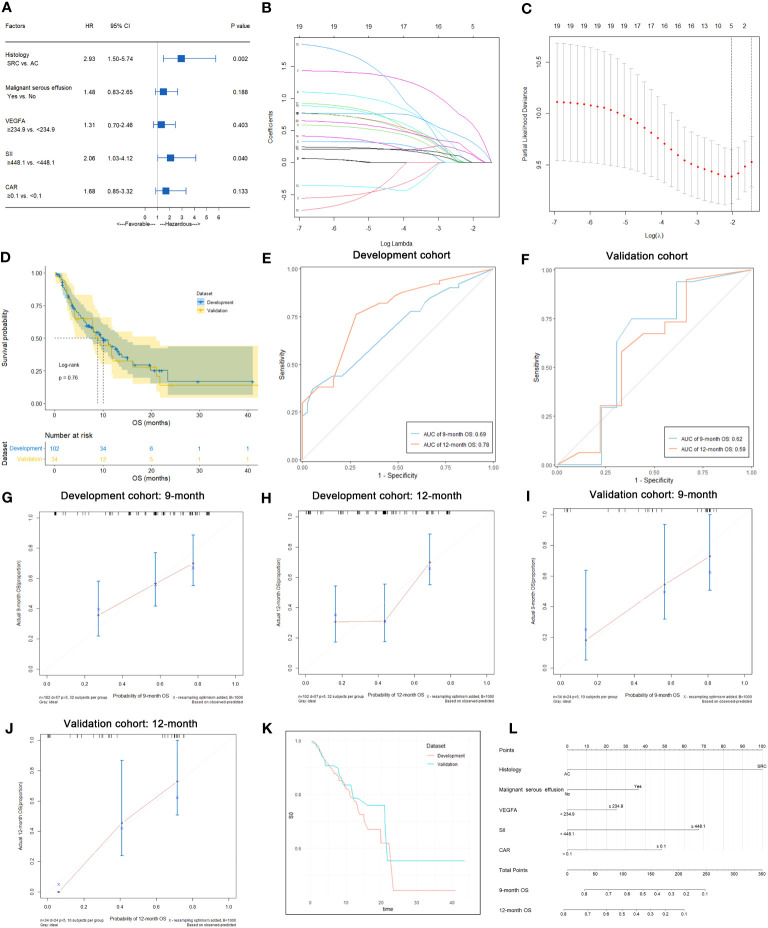
Predictive variable screening and construction, evaluation and presentation of the OS predictive model in HER2-negative AGC patients. **(A)** Forest plot showing the multivariate Cox analysis of OS with the predictive variables that were significantly associated with OS in the univariate Cox analyses. **(B, C)** Predictive variables were screened using the LASSO regression model via 10-fold cross-validation based on minimum criteria. The dotted vertical line in C was plotted at the value selected using 10-fold cross-validation in C. **(D)** Kaplan−Meier survival curves of OS of HER2-negative AGC patients in the development and validation cohorts. **(E)** Time-dependent ROC curves of the OS predictive model at 9 and 12 months for HER2-negative AGC in the development cohort. **(F)** Time-dependent ROC curves of the OS predictive model at 9 and 12 months for HER2-negative AGC in the validation cohort. **(G, H)** Calibration plot of the OS predictive model at 9 and 12 months for HER2-negative AGC in the development cohort. **(I, J)** Calibration plot of the OS predictive model at 9 and 12 months for HER2-negative AGC in the validation cohort. **(K)** Baseline survival curves of OS of HER2-negative AGC patients in the development and validation cohorts. **(L)** Nomogram to predict the probability of 9-month or 12-month OS in patients with HER2-negative AGC.

In this study, 57 of 102 patients without loss to follow-up reached the OS endpoint. As shown in **Table S4**, univariate analysis suggested that histology (p<0.001), malignant serous effusion (p=0.007), VEGFA (p=0.008), SII (p=0.003) and CAR (p=0.010) were significantly associated with OS in patients with HER2-negative AGC. Multivariate analysis indicated that histology (signet-ring cell carcinoma vs. adenocarcinoma, HR: 2.93, 95% CI: 1.50-5.74, p=0.002) and SII (≥448.1 vs. <448.1, HR: 2.06, 95% CI: 1.03-4.12, p=0.040) were independent prognostic factors for OS in patients with HER2-negative AGC (**Table S4**; **Figure 7A**). Kaplan−Meier plots of patients stratified by each independent prognostic factor are shown in **Figures 6E, F**.

### 3.10 Development of nomograms for predicting the OS of HER2-negative AGC patients

In 102 HER2-negative AGC patients with complete follow-up data, variables were screened according to the LASSO Cox regression model (**Figures 7B, C**), clinical significance, and recommendations from oncology experts, and histology, malignant serous effusion, VEGFA, SII and CAR were selected to fit a Cox regression model for predicting OS that met the proportional hazards assumption. The mOS was similar between the development and validation cohorts (p=0.760, **Figure 7D**). The model had Harrell’s C-indexes of 0.69 and 0.66 after calibration with internal validation using bootstrap resampling (1,000 repetitions) in the development cohort and 0.62 in the validation cohort. The time-dependent ROC curves and calibration curves of the 9-month and 12-month OS prediction in the development (AUC: 0.69 and 0.78, respectively) and validation cohorts (AUC: 0.62 and 0.59, respectively) are illustrated in **Figures 7E–J**. The baseline survival curves of OS were similar in both development and validation cohorts (**Figure 7K**). The nomogram and web-based dynamic nomogram of the predictive OS model are presented in **Figure 7L** and https://cancerprediction.shinyapps.io/os_prediction_of_her2-agc/. The calculation formula for the OS model is presented on the webpage of the dynamic nomogram.

## 4 Discussion

HER2-negative GC, which lacks precision medical care, is the main type of GC and has a poor prognosis. Establishment of predictive models can help effectively stratify populations and optimize individualized chronic disease management strategies for this malignancy. In this study, the role of immune-inflammatory biomarkers including serum VEGFA and SII in the treatment response and survival of HER2-negative AGC patients was clarified by a cohort study, and predictive nomograms related to therapeutic efficacy and prognosis prediction were established. Internal and external validation has shown that these nomograms based on immune-inflammatory indicators perform well in predicting the treatment response and survival of HER2-negative AGC patients and will provide an important reference for the clinical management of this lethal disease.

VEGF is a family of pivotal growth factors and signaling molecules involved in angiogenesis. Binding to its receptor, VEGFR, activates a complex cascade of downstream signaling pathways that leads to neovascularization and vasodilation (31). Inhibition of VEGF signaling activity impairs these pathways, resulting in a reduction in tumor proliferation and invasion in GC (32). As a major member of the VEGF family, VEGFA has been reported to be associated with prognosis in various malignancies (10, 11) and is considered to be a useful biomarker of the progression and remission of diseases, including GC (33). Clinically, ramucirumab is the primary antiangiogenic agent used for AGC. Ramucirumab was confirmed as the preferred agent for second-line and beyond therapy in AGC by the REGARD (34) and RAINBOW (35) studies, but the failure of the RAINFALL (36) study prevented ramucirumab from entering first-line therapy. One reason for these findings may be that the combination of ramucirumab/fluoropyrimidine/cisplatin may not be the optimal regimen; however, the lack of a screened population of patients predicted to benefit from antiangiogenic therapy may also contribute to the failure of the study. To investigate the potential role of serum VEGFA in screening the population that may benefit from chemotherapy, we previously launched a prospective cohort study in highly malignant SCLC and revealed the unique ability of VEGFA to predict first-line chemotherapeutic efficacy and survival in patients with this kind of disease (37). In this previous study, we also found that the median VEGFA level decreased after 2 cycles of chemotherapy and returned to pretreatment levels at progression, but the changes in VEGFA were not associated with therapeutic efficacy and PFS (37). Therefore, we conducted a follow-up study of AGC patients and focused on investigating the role of VEGFA at baseline. The results showed that VEGFA was not only an independent predictor of first-line chemotherapy efficacy and corresponding PFS outcomes but also held the greatest weight in the therapeutic efficacy and PFS models. Despite its reduced value for OS prediction, it still plays a role in the OS predictive model. PFS reflects the survival benefit of stage-specific antitumor therapies. Since patients with AGC in China often receive 3 or more lines of antitumor therapy (38), including late-line chemotherapy and antiangiogenic targeted therapy, the benefit in terms of PFS after first-line therapy does not reliably reflect the benefit in terms of OS, which could be why baseline VEGFA did not predict OS in this study. Therefore, for HER2-negative AGC, VEGFA can be used as a biomarker for evaluating efficacy and short-term survival but is a weak predictor of long-term survival. VEGFA-based nomograms would help clinicians screen chemotherapy-resistant populations in advance and provide a basis for stratifying patients for future intensive therapy studies.

It is noted that the present study identified SII as an independent prognostic factor for PFS and OS after first-line chemotherapy in patients with HER2-negative AGC, and a model including the SII was effective in predicting patient OS. The SII considers the abundance of lymphocytes that play a major role in inducing cytotoxic cell death and suppressing cancer cell proliferation (39), neutrophils that are known to secrete a variety of cytokines (VEGF, IL-8, IL-16, etc.) that stimulate tumor cell growth and metastasis (40), and platelets that can promote cancer cell arrest at the endothelium and support the establishment of secondary lesions of cancer cells (41). Therefore, the elevation of the SII may reflect cancer-promoting activity in the tumor microenvironment (TME), which eventually leads to poor prognosis (42, 43). The predictive effect of the SII on PFS and OS in the present study supports the notion that the immune inflammatory response broadly affects antitumor therapy and ultimately affects the survival of patients both in the short and long term. Currently, immune checkpoint inhibition has become a new standard treatment of AGC. Results from KEYNOTE-063 (44) and JAVELIN Gastric 100 (45) studies have confirmed that ICIs monotherapies failed to outperform the benefit from chemotherapy in patients with AGC. In contrast, ICIs combined with chemotherapy explored in CheckMate-649 (46) and ORIENT-16 (47) studies significantly improved the response rate and survival and has become the first-line standard treatment for this kind of patients, which implied that chemotherapy remains the cornerstone of systemic treatment for this fatal disease. Chemotherapy has been validated as potent immunogenic cell death (ICD) inducer which can destruct cancer cells and help exposing immunostimulatory molecules like damage-associated molecular patterns (DAMPs) and cytokines like type I interferons (IFNs), and thus increase the sensitivity of tumor to ICIs (48). In addition, in our study focus on patients with chemotherapy, VEGFA, SII and CAR in the predictive models are also immune-inflammatory indicators. Therefore, the determination of the sensitivity of chemotherapy with our models may contribute to the judgment of the efficacy of immunotherapy and this needs to be validated in future study on patients receiving ICIs.

Most importantly, novel immune-inflammation-based clinical predictive nomograms in HER2-negative AGC were developed and validated in this study. Most of the previous prediction models were based on retrospective studies and mainly focused on diagnosis (49) or prediction of neoadjuvant chemotherapy response (50) or survival in GC (51). Models in the present study were based on a prospective cohort study with more reliable information and less recall bias than previous studies. Validation based on an independent cohort enhanced the generalizability of the study results. Moreover, this study not only established a satisfactory prediction model for OS but also innovatively developed predictive models for first-line chemotherapy efficacy and PFS, providing new approaches for the screening of primary chemotherapy-resistant populations. In addition, compared with previous models, this study added new blood indicators, such as VEGFA, SII, and other traditional markers, making the results more comprehensive, reliable, and clinically applicable.

This study had some limitations. As this was a single-center study, the sample size was relatively small, which limited the number of variables that could be included in the models, leading to a decrease in their predictive power. In addition, although we enrolled HER2-negative AGC patients for external validation, data from other institutions would allow a more robust evaluation of the universality of the predictive models, and this will be complemented by a subsequent validation study with multicenter data.

In summary, this study established novel and effective series of immune-inflammation-based predictive models that showed favorable performance in evaluating first-line chemotherapeutic efficacy and survival in HER2-negative AGC patients. Nomograms constructed using VEGFA, SII, and other accepted clinicopathological variables could be utilized as an efficient computational technique for predicting the clinical prognosis of this kind of patients. We considered that angiogenesis and immune inflammation are interlinked in the treatment of GC and collectively affect the prognosis of patients with AGC. In the future, the use of reliable predictive models to screen patients who may benefit or not benefit from specific treatment may lead to the broad implementation of individualized medicine.

## Data availability statement

The raw data supporting the conclusions of this article will be made available by the authors, without undue reservation.

## Ethics statement

The studies involving human participants were reviewed and approved by Institutional Review Board of the First Affiliated Hospital of Bengbu Medical College. The patients/participants provided their written informed consent to participate in this study.

## Author contributions

Conceptualization, JY. Methodology, YY and YS. Software, YY and YS. Formal Analysis, YY, YS and HY. Investigation, JW, QC, YL, JL and YZ. Resources, ZZ, MW. Data Curation, JW. Writing-Original Draft Preparation, YY and YS. Writing-Review and Editing, BJ, JY. Supervision, JY, YY. Project Administration, JY, YY. Funding Acquisition, YY. All authors contributed to the article and approved the submitted version.
