# Supplementary material for: Development and validation of novel immune-inflammation-based clinical predictive nomograms in HER2-negative advanced gastric cancer
Source: Front Oncol. 2023 Sep 6;13:1185240. doi: 10.3389/fonc.2023.1185240 (PMC10516559; doi:10.3389/fonc.2023.1185240)
Supplement: Supplementary file 1 [file DataSheet_1.docx]

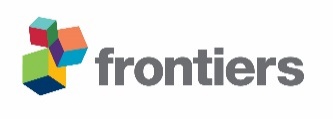
Supplementary Material

**Development and Validation of Novel Immune-Inflammation-Based Clinical Predictive Nomograms in HER2-Negative Advanced Gastric Cancer**

**Yan Yang^1,^** ^†^**, Yu Shao^1,^** ^†^**, Junjun Wang^2^, Qianqian Cheng^1^, Hanqi Yang^3^, Yulong Li^4^, Jing Liu^1^, Yangyang Zhou^1^, Zhengguang Zhou^1^, Mingxi Wang^1^, Baoan Ji^5^, Jinghao Yao^1, 6,^ ***

***Correspondence:** Jinghao Yao: leijiyasi@outlook.com

# Supplementary figure

# Supplementary tables


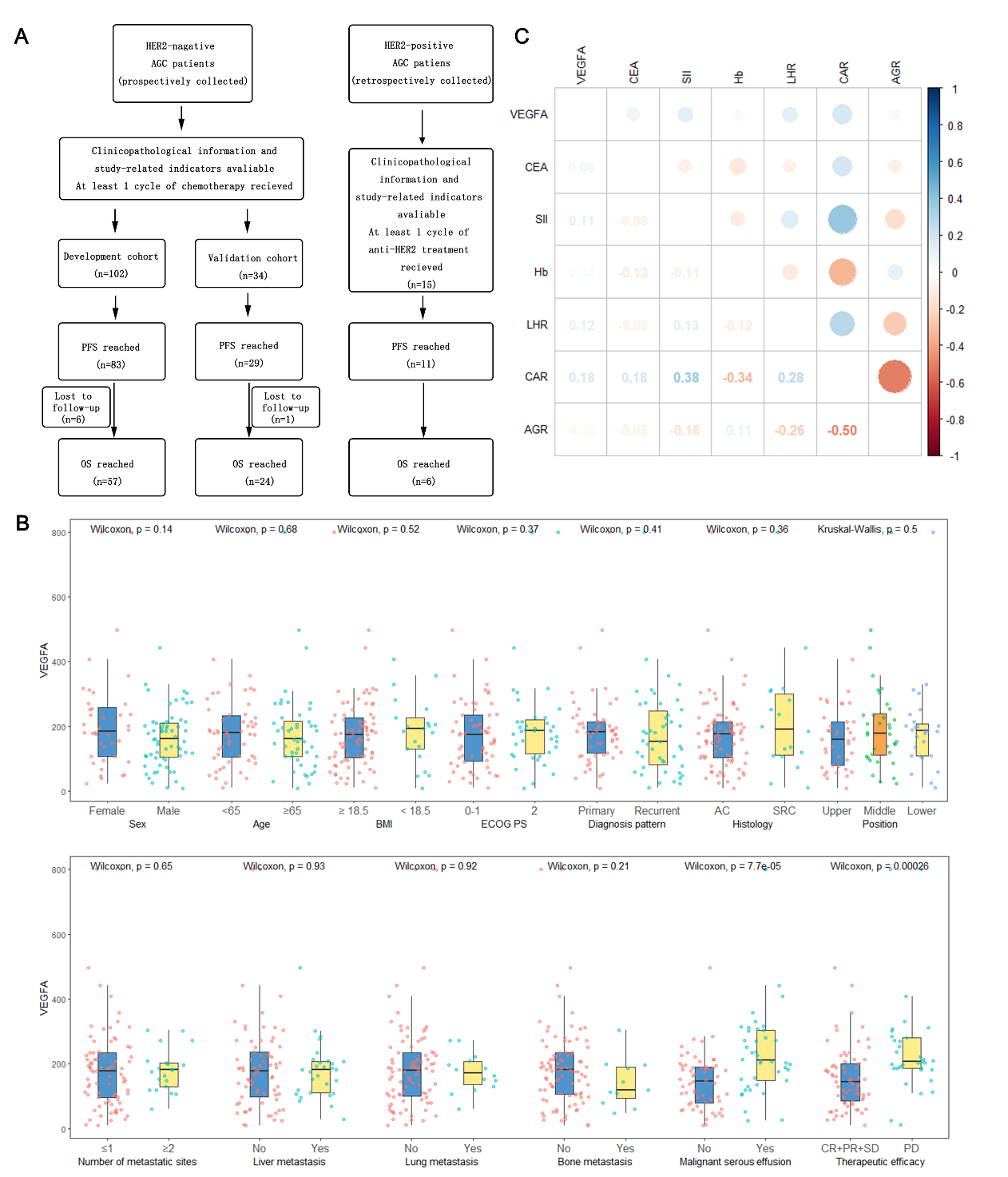


**Figure S1.** The study design and relationships between baseline serum VEGFA and the clinicopathological indicators. (A) The study flowchart. (B) The relationships between baseline serum VEGFA and the clinicopathological characteristics of HER2-negative AGC patients. (C) Heatmap of correlations between different laboratory indicators.

| **Table S1.** Relationship between baseline serum VEGFA and clinicopathological characteristics of HER2-positive AGC patients | | | | | |
| --- | --- | --- | --- | --- | --- |
| **Characteristics** | **Groups** | **HER2-positive cohort** | | | |
|  |  | **N (%)** | **Median** | **Statistics** | **p*-*value** |
| Sex | Female | 5 (33.3%) | 239.71 (102.98-264.50) | 28 | 0.768 |
|  | Male | 10 (66.7%) | 155.82 (107.67-253.50) |  |  |
| Age | <65 | 11 (73.3%) | 230.54 (113.30-262.82) | 28 | 0.489 |
|  | ≥65 | 4 (26.7%) | 126.72 (99.37-188.71) |  |  |
| BMI (kg/m^2^) | ≥ 18.5 | 12 (80.0%) | 137.36 (101.90-232.83) | 1 | 0.009** |
|  | < 18.5 | 3 (20.0%) | 318.48 (291.49-433.35) |  |  |
| ECOG PS | 0-1 | 12 (80.0%) | 142.07 (101.90-245.07) | 8 | 0.180 |
|  | 2 | 3 (20.0%) | 264.50 (207.80-406.36) |  |  |
| Diagnosis pattern | Primary | 11 (73.3%) | 151.10 (102.66-250.43) | 17 | 0.571 |
|  | Recurrent | 4 (26.7%) | 212.51 (145.54-278.00) |  |  |
| Histology | AC | 15 (13.3%) | - | - | - |
|  | SRC | 0 (0.0%) | - |  |  |
| Position | Upper | 2 (13.3%) | 112.10(106.33-117.86) | 2.89 | 0.236 |
|  | Middle | 7 (46.7%) | 102.98 (96.39-270.62) |  |  |
|  | Lower | 6 (40.0%) | 245.84 (178.03-263.66) |  |  |
| Number of metastatic sites | ≤1 | 11 (73.3%) | 151.10 (102.66-245.84) | 17 | 0.571 |
|  | ≥2 | 4 (26.7%) | 252.11 (202.18-278.00) |  |  |
| Liver metastasis | No | 8 (53.3%) | 113.30 (101.90-170.96) | 15 | 0.152 |
|  | Yes | 7 (46.7%) | 261.15 (200.12-283.02) |  |  |
| Lung metastasis | No | 10 (66.7%) | 195.54 (101.17-291.43) | 26 | 0.953 |
|  | Yes | 5 (33.3%) | 151.10 (123.62-239.71) |  |  |
| Bone metastasis | No | 12 (80.0%) | 195.54 (118.46-273.76) | 29 | 0.136 |
|  | Yes | 3 (20.0%) | 90.44 (90.01-165.07) |  |  |
| Malignant serous effusion^#^ | No | 14 (93.3%) | 155.82 (102.51-255.79) | 1 | 0.267 |
|  | Yes | 1 (6.7%) | 318.48 (318.48-318.48) |  |  |
| Therapeutic efficacy | CR+PR+SD | 13 (86.7%) | 151.10 (102.35-261.15) | 6 | 0.305 |
|  | PD | 2 (13.3%) | 274.51 (252.52-296.50) |  |  |

Notes: **: p<0.01.

^#^ Malignant serous effusion was diagnosed based on ascites cytology or clinical symptoms if abdominocentesis was contraindicated or cytology result was unavailable.

| **Table S2.** Univariate and multivariate Logistic regression analyses of the first-line therapeutic efficacy in HER2-negative AGC patients | | | | | | |
| --- | --- | --- | --- | --- | --- | --- |
| **Characteristics** | **Univariate** | | | **Multivariate** | | |
|  | **OR** | **95%CI** | **p-value** | **OR** | **95%CI** | **p-value** |
| Sex |  |  |  |  |  |  |
| Male vs. Female | 0.82 | 0.35-1.91 | 0.646 |  |  |  |
| Age |  |  |  |  |  |  |
| ≥65 vs. <65 | 0.91 | 0.40-2.09 | 0.822 |  |  |  |
| BMI (kg/m^2^) |  |  |  |  |  |  |
| <18.5 vs. ≥18.5 | 2.95 | 1.10-7.91 | 0.032* | 1.96 | 0.59-6.53 | 0.273 |
| ECOG PS |  |  |  |  |  |  |
| 2 vs. 0-1 | 1.56 | 0.67-3.64 | 0.301 |  |  |  |
| Diagnosis pattern |  |  |  |  |  |  |
| Recurrent vs. Primary | 1.47 | 0.64-3.37 | 0.369 |  |  |  |
| Histology |  |  |  |  |  |  |
| SRC vs. AC | 2.50 | 0.89-7.06 | 0.084 |  |  |  |
| Position |  |  |  |  |  |  |
| Middle vs. Upper | 0.85 | 0.30-2.37 | 0.754 |  |  |  |
| Lower vs. Upper | 2.36 | 0.85-6.61 | 0.101 |  |  |  |
| Number of metastatic sites |  |  |  |  |  |  |
| ≥2 vs. ≤1 | 2.29 | 0.86-6.13 | 0.098 |  |  |  |
| Liver metastasis |  |  |  |  |  |  |
| Yes vs. No | 1.14 | 0.46-2.85 | 0.772 |  |  |  |
| Lung metastasis |  |  |  |  |  |  |
| Yes vs. No | 2.38 | 0.76-7.48 | 0.136 |  |  |  |
| Bone metastasis |  |  |  |  |  |  |
| Yes vs. No | 1.22 | 0.33-4.51 | 0.764 |  |  |  |
| Malignant serous effusion^#^ |  |  |  |  |  |  |
| Yes vs. No | 3.29 | 1.39-7.79 | 0.007** | 2.08 | 0.72-5.99 | 0.173 |
| VEGFA (ng/L) |  |  |  |  |  |  |
| ≥180.2 vs. <180.2 | 9.00 | 3.26-24.87 | <0.001*** | 6.95 | 2.30-20.99 | 0.001** |
| CEA (μg/L) |  |  |  |  |  |  |
| ≥5.4 vs. <5.4 | 2.56 | 1.09-6.04 | 0.031* | 2.71 | 0.96-7.66 | 0.059 |
| SII (*10^9^) |  |  |  |  |  |  |
| ≥372.5 vs. <372.5 | 3.80 | 1.04-13.93 | 0.044* | 2.84 | 0.58-14.04 | 0.200 |
| Hb (g/L) |  |  |  |  |  |  |
| <90 vs. ≥90 | 1.28 | 0.45-3.62 | 0.643 |  |  |  |
| LHR |  |  |  |  |  |  |
| ≥1.7 vs. <1.7 | 3.26 | 0.69-15.52 | 0.137 |  |  |  |
| CAR (*10^-3^) |  |  |  |  |  |  |
| ≥0.4 vs. <0.4 | 3.01 | 1.26-7.18 | 0.013* | 1.86 | 0.65-5.32 | 0.245 |
| AGR |  |  |  |  |  |  |
| <1.5 vs. ≥1.5 | 1.00 | 0.41-2.41 | 1.000 |  |  |  |

Notes: *: p<0.05, **: p<0.01, ***: p<0.001.

^#^ Malignant serous effusion was diagnosed based on ascites cytology or clinical symptoms if abdominocentesis was contraindicated or cytology result was unavailable.

| **Table S3.** Univariate and multivariate Cox regression analyses of the first-line PFS in HER2-negative AGC patients | | | | | | |
| --- | --- | --- | --- | --- | --- | --- |
| **Characteristics** | **Univariate** | | | **Multivariate** | | |
|  | **HR** | **95%CI** | **p-value** | **HR** | **95%CI** | **p-value** |
| Sex |  |  |  |  |  |  |
| Male vs. Female | 0.82 | 0.53-1.27 | 0.381 |  |  |  |
| Age |  |  |  |  |  |  |
| ≥65 vs. <65 | 0.81 | 0.52-1.24 | 0.328 |  |  |  |
| BMI (kg/m^2^) |  |  |  |  |  |  |
| <18.5 vs. ≥18.5 | 1.64 | 0.97-2.77 | 0.066 |  |  |  |
| ECOG PS |  |  |  |  |  |  |
| 2 vs. 0-1 | 1.16 | 0.75-1.81 | 0.501 |  |  |  |
| Diagnosis pattern |  |  |  |  |  |  |
| Recurrent vs. Primary | 1.61 | 1.04-2.48 | 0.033* | 2.34 | 1.43-3.85 | 0.001** |
| Histology |  |  |  |  |  |  |
| SRC vs. AC | 2.19 | 1.29-3.70 | 0.004** | 1.88 | 1.07-3.3 | 0.027* |
| Position |  |  |  |  |  |  |
| Middle vs. Upper | 1.47 | 0.88-2.44 | 0.143 |  |  |  |
| Lower vs. Upper | 1.68 | 0.96-2.93 | 0.070 |  |  |  |
| Number of metastatic sites |  |  |  |  |  |  |
| ≥2 vs. ≤1 | 1.62 | 0.98-2.69 | 0.062 |  |  |  |
| Liver metastasis |  |  |  |  |  |  |
| Yes vs. No | 1.11 | 0.70-1.76 | 0.663 |  |  |  |
| Lung metastasis |  |  |  |  |  |  |
| Yes vs. No | 1.65 | 0.91-3.00 | 0.101 |  |  |  |
| Bone metastasis |  |  |  |  |  |  |
| Yes vs. No | 0.99 | 0.51-1.92 | 0.980 |  |  |  |
| Malignant serous effusion^#^ |  |  |  |  |  |  |
| Yes vs. No | 1.98 | 1.27-3.08 | 0.003** | 1.39 | 0.85-2.27 | 0.187 |
| VEGFA (ng/L) |  |  |  |  |  |  |
| ≥179.1 vs. <179.1 | 2.35 | 1.52-3.65 | <0.001*** | 2.49 | 1.51-4.11 | <0.001*** |
| CEA (μg/L) |  |  |  |  |  |  |
| ≥5.3 vs. <5.3 | 1.49 | 0.97-2.3 | 0.069 |  |  |  |
| SII (*10^9^) |  |  |  |  |  |  |
| ≥376.9 vs. <376.9 | 2.21 | 1.25-3.88 | 0.006** | 1.98 | 1.10-3.56 | 0.023* |
| Hb (g/L) |  |  |  |  |  |  |
| <90 vs. ≥90 | 1.12 | 0.66-1.91 | 0.676 |  |  |  |
| LHR |  |  |  |  |  |  |
| ≥3.9 vs. <3.9 | 1.60 | 0.88-2.92 | 0.126 |  |  |  |
| CAR (*10^-3^) |  |  |  |  |  |  |
| ≥0.3 vs. <0.3 | 1.57 | 1.01-2.45 | 0.045* | 1.52 | 0.95-2.44 | 0.083 |
| AGR |  |  |  |  |  |  |
| <1.5 vs. ≥1.5 | 1.01 | 0.64-1.60 | 0.975 |  |  |  |

Notes: *: p<0.05, **: p<0.01, ***: p<0.001.

^#^ Malignant serous effusion was diagnosed based on ascites cytology or clinical symptoms if abdominocentesis was contraindicated or cytology result was unavailable.

| **Table S4.** Univariate and multivariate Cox regression analyses of OS in HER2-negative AGC patients | | | | | | |
| --- | --- | --- | --- | --- | --- | --- |
| **Characteristics** | **Univariate** | | | **Multivariate** | | |
|  | **HR** | **95%CI** | **p-value** | **HR** | **95%CI** | **p-value** |
| Sex |  |  |  |  |  |  |
| Male vs. Female | 0.79 | 0.46-1.33 | 0.369 |  |  |  |
| Age |  |  |  |  |  |  |
| ≥65 vs. <65 | 0.92 | 0.54-1.57 | 0.765 |  |  |  |
| BMI (kg/m^2^) |  |  |  |  |  |  |
| <18.5 vs. ≥18.5 | 1.56 | 0.84-2.93 | 0.162 |  |  |  |
| ECOG PS |  |  |  |  |  |  |
| 2 vs. 0-1 | 1.16 | 0.68-1.97 | 0.579 |  |  |  |
| Diagnosis pattern |  |  |  |  |  |  |
| Recurrent vs. Primary | 1.45 | 0.86-2.44 | 0.163 |  |  |  |
| Histology |  |  |  |  |  |  |
| SRC vs. AC | 3.37 | 1.81-6.28 | <0.001*** | 2.93 | 1.50-5.74 | 0.002** |
| Position |  |  |  |  |  |  |
| Middle vs. Upper | 1.12 | 0.60-2.09 | 0.729 |  |  |  |
| Lower vs. Upper | 1.41 | 0.73-2.71 | 0.309 |  |  |  |
| Number of metastatic sites |  |  |  |  |  |  |
| ≥2 vs. ≤1 | 1.53 | 0.83-2.82 | 0.170 |  |  |  |
| Liver metastasis |  |  |  |  |  |  |
| Yes vs. No | 1.39 | 0.80-2.40 | 0.241 |  |  |  |
| Lung metastasis |  |  |  |  |  |  |
| Yes vs. No | 1.42 | 0.69-2.91 | 0.344 |  |  |  |
| Bone metastasis |  |  |  |  |  |  |
| Yes vs. No | 0.99 | 0.44-2.22 | 0.984 |  |  |  |
| Malignant serous effusion^#^ |  |  |  |  |  |  |
| Yes vs. No | 2.05 | 1.21-3.48 | 0.007** | 1.48 | 0.83-2.65 | 0.188 |
| VEGFA (ng/L) |  |  |  |  |  |  |
| ≥234.9 vs. <234.9 | 2.17 | 1.22-3.85 | 0.008** | 1.31 | 0.70-2.46 | 0.403 |
| CEA (μg/L) |  |  |  |  |  |  |
| ≥69.5 vs. <69.5 | 1.37 | 0.72-2.60 | 0.331 |  |  |  |
| SII (*10^9^) |  |  |  |  |  |  |
| ≥448.1 vs. <448.1 | 2.66 | 1.40-5.05 | 0.003** | 2.06 | 1.03-4.12 | 0.040* |
| Hb (g/L) |  |  |  |  |  |  |
| <90 vs. ≥90 | 1.56 | 0.85-2.86 | 0.153 |  |  |  |
| LHR |  |  |  |  |  |  |
| ≥1.5 vs. <1.5 | 2.75 | 0.83-9.10 | 0.097 |  |  |  |
| CAR (*10^-3^) |  |  |  |  |  |  |
| ≥0.1 vs. <0.1 | 2.33 | 1.23-4.43 | 0.010* | 1.68 | 0.85-3.32 | 0.133 |
| AGR |  |  |  |  |  |  |
| <1.5 vs. ≥1.5 | 1.59 | 0.88-2.88 | 0.124 |  |  |  |

Notes: *: p<0.05, **: p<0.01, ***: p<0.001.

^#^ Malignant serous effusion was diagnosed based on ascites cytology or clinical symptoms if abdominocentesis was contraindicated or cytology result was unavailable.
